# Advances in bioinformatics and multi-omics integration: transforming viral infectious disease research in veterinary medicine

**DOI:** 10.1186/s12985-025-02640-x

**Published:** 2025-01-31

**Authors:** Alyaa Elrashedy, Walid Mousa, Mohamed Nayel, Akram Salama, Ahmed Zaghawa, Ahmed Elsify, Mohamed E. Hasan

**Affiliations:** 1 Department of Animal Medicine and Infectious Diseases, Faculty of Veterinary Medicine, University of Sadat City, Sadat, Egypt; 2 Bioinformatics Department, Genetic Engineering and Biotechnology Research Institute, University of Sadat City, Sadat, Egypt

**Keywords:** Genomics, Proteomics, NGS, Transcriptomics, Metabolomics, Metagenomics

## Abstract

The world is changing due to factors like bioterrorism, massive environmental changes, globalization of trade and commerce, growing urbanization, changing climate, and pollution. Numerous diseases have emerged because of these factors, especially in companion and food-producing animals. Numerous pathogens have established themselves in naïve populations, harming reproduction, productivity, and health. Bioinformatics is considered a valuable tool in infectious disease research, as it provides a comprehensive overview of the identification of pathogens, their genetic makeup, and their evolutionary relationship. Therefore, there is an urgent need for a novel bioinformatics approach to help decipher and model viral epidemiology and informatics on domestic animals and livestock. With significant advancements in bioinformatics and NGS, researchers can now identify contigs, which are contiguous sequences of DNA that are assembled from overlapping fragments, assemble a complete genome, perform phylogenetic analysis to diagnose, investigate the risk of viral diseases in animals, handle and share large biological datasets across various species. Additionally, multi-omics data integration further deepens our understanding of homology, divergence, mutations, and evolutionary relationships, providing a comprehensive perspective on the molecular mechanisms driving animal pathogens infections. This review aims to reveal the importance of utilizing the multidisciplinary areas of bioinformatics, genomics, proteomics, transcriptomics, metabolomics, and metagenomics and their roles in studying viral infectious diseases in veterinary medicine that will eventually improve the health of animals.

## Background

Bioinformatics is essential in infectious disease research. It helps identify and understand pathogens, their genetic makeup, and how they evolve and spread. This knowledge is crucial for creating diagnostic tools, vaccines, and targeted treatments [1]. With advancements in bioinformatics and NGS, collecting and analyzing large amounts of biological data from various species has become easier and more efficient. This field covers several important areas: genomics, proteomics, transcriptomics, and metabolomics, each providing unique insights into the intricacy of biological systems [2]. Genomics involves the comprehensive analysis of an organism’s entire genetic material, helping to identify genes and regulatory elements and understand their roles in health and disease [3]. Proteomics is about studying proteins on a large scale, including their structures, functions, and interactions, which gives crucial insights into how cells work [4]. Transcriptomics looks at all the RNA transcripts produced by the genome, providing a clear picture of gene expression patterns and regulatory mechanisms [5]. Metabolomics examines the chemical processes involving metabolites, giving a detailed understanding of the metabolic state of a cell or organism [6]. Multi-omics integration provides insights into homology, divergence, mutations, and evolutionary relationships, helping to understand how structures and functions change over time.

Tracking how viruses evolve and spread has become a cornerstone of disease control and prevention. Through bioinformatics tools, researchers can uncover critical insights into the mechanisms of viral adaptation and interspecies transmission [7, 8]. These insights guide public health strategies by identifying variants of concern early, enabling timely interventions. Similarly, in veterinary science, tracking evolutionary trends in viral pathogens supports the timely development of vaccines and diagnostics approaches.

For example, FMDV, avian influenza, and ASFV are among the most extensively studied pathogens using multi-omics approaches due to their global significance and economic impact. FMDV’s high mutation rate requires continuous genomic monitoring to ensure vaccine effectiveness and limit outbreaks [9–11]. Avian influenza’s zoonotic potential and rapid evolution highlight the importance of transcriptomics and proteomics in understanding viral-host interactions and developing cross-species vaccines [12]. ASFV’s devastating effects on swine production have driven transcriptomics research to uncover the different susceptibility on the pigs [5]. Additionally, transcriptomic analyses of viral infections like BVD and rabies virus have identified immune pathways and gene networks activated during infection, offering potential targets for therapeutic interventions [13–15]. Transcriptomics also enhances this understanding by revealing host-pathogen interactions at the gene expression level in NDV [16].

Stimulatingly, metagenomic studies have been instrumental in detecting newly emerging viral species in livestock, enabling the early identification of threats such as LSDV [17]. Proteomics complements this by mapping viral and host protein interactions, shedding light on critical processes such as viral entry and replication as seen in LSDV studies, where proteomic data has informed vaccine evaluation [18, 19]. Similarly, metabolomics highlights how viral infections reprogram host metabolism that emphasize the role of metabolic pathways in disease progression and biomarker discovery like in MDV [20].

Despite these achievements, challenges such as high costs, computational demands, and data integration complexities remain difficulties in application of such technologies. Addressing these issues requires innovative solutions and collaborative efforts to develop standardized workflows and accessible databases [21]. Together, multi-omics integration provides a holistic, systems-level understanding of veterinary viral diseases, accelerating advancements in diagnostics, vaccine development, and therapeutics [22]. These efforts not only improve animal health and welfare but also contribute to global food security by safeguarding economically important livestock and companion animals. Therefore, this review aims to reveal the importance of utilizing the multidisciplinary areas of bioinformatics, genomics, proteomics, transcriptomics, metabolomics, and metagenomics and their roles in studying viral infectious diseases in veterinary medicine that will eventually improve the health of animals.

## Genomics application of different veterinary viral infectious diseases

### Genome sequencing

Genome sequencing is crucial in virology because it provides a comprehensive overview of the virus’s genetic structure, evolution, diversity, and the causation of the diseases. As many viruses mutate quickly, continuous genome monitoring is mandatory to keep track of these changes and prevent the emergence of new virus strains with distinct properties, such as increased virulence, lower transmission rates, or drug resistance [23]. Moreover, it plays a key role in epidemiological insights through tracking the transmission pathways and identifying sources of outbreaks. This helps to understand the viral spread dynamics and informs public health responses to implement control measures instantly [24]. Also, it has a role in knowing host-virus interactions to understand the immune evasion, mechanisms of infection, and disease progression [25, 26]. For instance, mutation analyses applied on SARS-CoV-2 structural proteins showed that the most common mutations were D614G in the spike protein, T9I in the envelope protein, I82T in the membrane protein, and R203M/R203K in the nucleocapsid protein. These mutations occurred in specific regions of each protein and varied geographically, with D614G being the most frequent globally. Such findings underscore the importance of monitoring mutations in structural proteins to develop more targeted drugs, vaccines, and diagnostics [7, 8].

WGS is a comprehensive technique that analyzes the entire genomic material of an organism in a single run. It encompasses all of the genome, including coding (exons) and non-coding (introns) regions [27]. The process begins with DNA extraction from a biological sample, then fragmented into smaller pieces. Adaptor sequences are added, and these sequences are read using HTS technologies such as Illumina or PacBio. Using bioinformatics tools, these short reads are assembled into a continuous sequence based on the reference genome [28]. Via the comprehensive genetic information provided with genome sequencing, it becomes easier to develop effective strategies for controlling and preventing viral infectious diseases, leading to better health outcomes for human and veterinary medicine.

#### Sanger sequencing

The first sequencing method is Sanger sequencing, known for its high accuracy compared to newer technologies. In this method, DNA is amplified using special chain-terminating molecules that stop DNA replication at various points. The resulting DNA fragments vary in length and are separated and analyzed using capillary electrophoresis. The final sequence is then detected using fluorescent tags on these fragments [29].

#### Next-generation sequencing

NGS revolutionized genome sequencing by enabling the simultaneous reading of millions of DNA fragments in a single run, making the process faster and more cost-effective [30]. It has different platforms such as Illumina, PacBio, and Ion Torrent, which play a crucial role in this HTS techniques [31]. This process starts with preparing the DNA library, where DNA is fragmented to different lengths and tagged with specified adaptors. That is followed by the amplification of the fragmented DNA into short sequence reads with varying sizes, depending on the platform used. Bioinformatics tools are then employed to assemble these sequence reads into a complete genome based on the reference genome [32].

#### Third-generation sequencing

Third-generation sequencing is often distinguished by its long-read sequencing methods and does not require the DNA to be amplified beforehand. It includes Oxford Nanopore and PacBio’s SMRT sequencing. For instance, with nanopore sequencing, single DNA molecules are threaded through tiny pores, and the changes in electrical current are used to measure the DNA sequence. However, the PacBio approach uses circular DNA templates and fluorescently labeled nucleotides, which are incorporated by DNA polymerase, while a real-time detection system captures the fluorescent signals [33].

#### RNA sequencing (RNA-Seq)

RNA-Seq is a powerful technique for analyzing entire RNA molecules produced from the transcription process. It is started by RNA extraction from a biological sample and then converted into cDNA. Next, this cDNA is sequenced using NGS technologies. RNA-Seq provides detailed insights into gene expression, alternative splicing events, and regulatory mechanisms. It reveals how genes are switched on or off in different conditions [34].

Each sequencing technology offers significant advantages for different genetic research objectives. WGS is noteworthy for its capacity to provide a comprehensive genomic profile, which is critical in viral research. This rigorous approach is critical for tracking mutations, producing vaccinations, and devising effective therapies, ensuring a complete grasp of the viruses under research.

### Processing of sequencing data

As previously stated, sequencing technologies often output raw data files containing millions of short reads. To be useful, this data must go through numerous processing steps. Each sequencing method has its own set of requirements and relies on certain bioinformatics tools to analyze the data and extract useful biological insights.

#### Quality control

Quality control is a vital first step in sequencing data analysis. Tools like FastQC and MultiQC are employed to evaluate the raw data, focusing on metrics such as sequence quality scores, read length distribution, and GC content [35]. The outcome is a set of quality reports that pinpoint and filter out low-quality reads. This process is crucial as it ensures that only reliable data is used in subsequent analysis, thereby improving the overall accuracy and dependability of the results.

#### Read trimming and filtering

The trimming and filtering step includes removing adapter sequences, trimming off low-quality bases, and discarding any short reads. Tools such as Trimmomatic and Cutadapt are used to clean up the sequencing data [36, 37]. The result is a refined dataset with only high-quality reads, which ensures that only reliable data is used in subsequent analyses. This step improves the overall accuracy and dependability of the results.

#### Read alignment/mapping

Read alignment, also known as mapping, uses tools like Bowtie2 [38], BWA [39], Minimap2 [40], and STAR [41] to match produced reads to a reference genome or to assemble them from scratch (Table 1). This process results in a collection of aligned reads in BAM or SAM format. This step is crucial because it sets the stage for identifying genetic variations and understanding the genomic context of the sequences. Accurate alignment is key for the reliable detection of variants and for making meaningful interpretations from the data.

#### Variant calling

In the variant calling step, we use tools like Samtools [42], FreeBayes [43], GATK [44], and VarScan [45], to find genetic variations, such as SNPs. This process generates a VCF, which lists all the genetic variants. This step is pivotal because it uncovers genetic differences that can significantly impact gene function or be associated with diseases.

#### Annotation

Annotation is the process of using tools like SnpEff [46], ANNOVAR [47], and VEP [48], to add functional details to the genetic variants. This step results in annotated VCF files and summary reports that link genetic variations to specific genes. It evaluates how these variations might impact protein function and identifies connections to known diseases.

#### Data visualization

Finally, data visualization tools like IGV [49], UCSC Genome Browser [50], and DNAPlotter [51] help create visual representations of the sequencing data. This step creates visual graphs and interactive plots, such as genome browser tracks and coverage maps. Visualization is key for interpreting and presenting the results, making complex data easier to understand. It helps researchers identify patterns, trends, and anomalies that might otherwise go unnoticed, providing clearer insights into the data.

**Table 1:** Concise overview of each step in the sequencing data processing workflow

Step	Key Tools	Link
Quality Control	FastQC, MultiQC	https://www.bioinformatics.babraham.ac.uk/projects/fastqc/ https://multiqc.info/
Read Trimming and Filtering	Trimmomatic, Cutadapt	http://www.usadellab.org/cms/?page=trimmomatic https://cutadapt.readthedocs.io/en/stable/
Read Alignment/Mapping	BWA, Bowtie2, STAR, Minimap2	https://bio-bwa.sourceforge.net/ https://bowtie-bio.sourceforge.net/bowtie2/index.shtml http://code.google.com/p/rna-star/. https://github.com/lh3/minimap2
Variant Calling	GATK, FreeBayes, Samtools	https://gatk.broadinstitute.org/hc/en-us https://github.com/freebayes/freebayes http://samtools.sourceforge.net
Annotation	ANNOVAR, SnpEff, VEP	http://www.openbioinformatics.org/annovar/. https://pcingola.github.io/SnpEff/ https://www.ensembl.org/info/docs/tools/vep/index.html
Data Visualization	IGV, UCSC Genome Browser	https://igv.org/ https://genome.ucsc.edu/

### Comparative genomic analysis

MSA is a key bioinformatics technique that aligns more than two DNA, RNA, or protein sequences to highlight their similarities. By comparing these sequences, MSA uncovers important functional, structural, or evolutionary relationships, helping us understand how they are connected. This process is also key for identifying homology, which refers to the similarities between sequences due to a common ancestor. Tools like Clustal Omega [52], MUSCLE [53], and MAFFT [54], are commonly used for MSA, each providing unique methods to create optimal alignments. MSA is crucial for identifying conserved regions, understanding sequence variation, and making functional predictions about genes and proteins.

Phylogenetic analysis is the study of evolutionary relationships among biological species based on genetic data. Therefore, building on the insights from MSA, phylogenetic analysis takes these aligned sequences and uses them to create a phylogenetic tree that can track epidemiological studies for viral outbreaks. This tree maps out how the sequences are related through evolution, showing the branching relationships and common ancestors. Tools such as MEGA 11 [55], PhyML [56], and RAxML [57], are typically employed to create and analyze these trees. Phylogenetic analysis is essential for unraveling the evolutionary history of species and tracking how diseases originate and spread. By identifying conserved and unique regions in genes or proteins, these techniques provide valuable insights that can guide the development of targeted treatments and vaccines [3].

Additionally, comparative analyses can be conducted using generic tools like ClustalW, BLAST, and Keyword Search algorithms [58]. From these search algorithms is ViroBLAST, a specialized tool for performing searches on viruses across multiple databases [59]. Also, the Alvira tool provides a graphical representation of the MSA of different comparative viral genomes [60]. Furthermore, researchers can conduct a comparative analysis of coronavirus traits and genetic material using the CoVDB database [61]. The specialized platform ViralZone is considered a repository for viral genome and proteome and gathers information on viral molecular biology, host interaction, classification, epidemiology, and structures supplied with high-resolution images [62]. The Simmonics program was upgraded to include the basic arrangement editor program package, allowing users to modify and verify provided arrangements and analyze progress for diversity and phylogeny [63]. Genetic modifications in viral genomes led to variations in their proteins, causing changes in viral characteristics like destructive abilities and interactions with hosts. The ViralORFeome database houses all viral ORF variations, mutants, and tools for ORF-specific cloning procedures [64].

### Genomics in different veterinary viral infections

Genome sequencing is essential for tackling viral diseases in both livestock and poultry. For instance, FMD, BVD, BTV, and Avian Influenza. For FMD, sequencing helps track the virus’s high mutation rate, identify new variants, and update vaccines to keep them effective [11, 65, 66]. Similarly, with BVD, sequencing provides insights into how the virus evolves and spreads, which is pivotal for developing effective control measures and vaccines [67, 68]. During outbreaks of the BTV, sequencing allows for real-time monitoring of viral changes, enabling prompt response to contain the disease [69]. Avian influenza viruses change rapidly, and specific genetic mutations influence their potential to infect mammals. Keeping a close watch on these changes and understanding how the virus spreads is crucial for effectively managing and preventing outbreaks in animals and humans [70]. Sharing this genomic data globally enhances collaboration and helps coordinate responses to emerging viral threats. For example, monitoring the ASFV through sequencing supports the development of better diagnostic tools and vaccines, ultimately helping manage and control these diseases more effectively [71].

For instance, new BTV variants have been identified worldwide, including in Australia, where ten serotypes have been found without causing illness. Phylogenetic analysis revealed that many Australian strains align with the eastern topotype while highlighting unique characteristics. The discovery of a new variant in Australia suggests that BTV-2 has recently arrived. Additionally, one Australian sample showed significant genetic differences from other local strains [72]. Another instance is a new Lyssavirus called Ikoma lyssavirus, which has been fully sequenced and identified as part of the Lyssavirus group, which includes rabies and several other serious infections. This virus, found in a Tanzanian African civet with rabies-like symptoms, is notable for being the most genetically distinct within its group [73]. Characterizing its genome is crucial, especially in understanding its genetic and antigenic differences compared to other Lyssaviruses. Despite the variety of lyssaviruses, existing antibodies generally offer protection against most species [74].

## Proteomics application of different veterinary viral infectious diseases

Proteomics is the study of proteins, focusing on their structure and function. A proteome is the complete set of proteins produced by an organism under specific conditions. Proteomics allows researchers to separate and characterize complex protein mixtures, identify proteins, determine their coding genes, and analyze post-translational modifications. This analysis is usually performed using MS after initial protein separation, with the method chosen based on the complexity of the proteome and the research goals [75]. High-throughput proteomics is highly effective for identifying protein profiles in animal body fluids, serving as a valuable diagnostic tool. It enables the discovery of overlooked fluids like saliva or tears and helps identify disease markers, which are crucial for diagnostics and developing new therapies [76]. Since proteins play various roles in biological systems, proteomics provides essential insights into disease mechanisms and aids in antiviral drug development during outbreaks. MS is key for accurately detecting and analyzing proteins, helping researchers understand protein interactions, signaling networks, and disease processes [77]. These approaches have played a significant role in enhancing our comprehension of cell signaling systems, revealing the intricate nature of protein-protein interactions in different cellular conditions, and improving our ability to diagnose diseases and gain a molecular understanding of their underlying mechanisms [78].

Furthermore, gel-based proteomics is commonly employed for protein characterization. 2-DE is a widely used gel-based proteomics method that separates proteins by their isoelectric point and molecular weight [79]. This technique involves separating proteins depending on their isoelectric point and molecular weight using polyacrylamide gels [80, 81]. Proteins that have been separated can be seen using staining techniques like Coomassie blue or silver staining and discovered through MS. This method offers benefits such as affordability, excellent clarity, and responsiveness [82, 83]. Currently, the primary constraint of 2-DE lies not in attaining ideal protein resolution but in its capacity to identify proteins. Additionally [84], demonstrated that proteomics presents a considerable challenge when it comes to identifying and quantifying proteins within intricate mixtures. Achieving this is possible by utilizing different bioinformatics tools like search engines to compare mass spectral data with protein sequence databases. Various tools and software for measuring proteins are accessible, including MaxQuant (www.maxquant.org), Proteome Discoverer (www.thermofisher.com), and Scaffold (https://support.proteomesoftware.com/hc/en-us). Moreover, there are various online databases where proteomics data can be stored and shared, such as ProteomeX Change (www.proteomexchange.org) and the GPMDB (https://thegpm.org ). These databases enable the merging of proteomic data from different origins. These databases allow proteomic data integration from various sources and facilitate the analysis and interpretation of proteomic data.

### Protein modelling

**Fig. 1:**
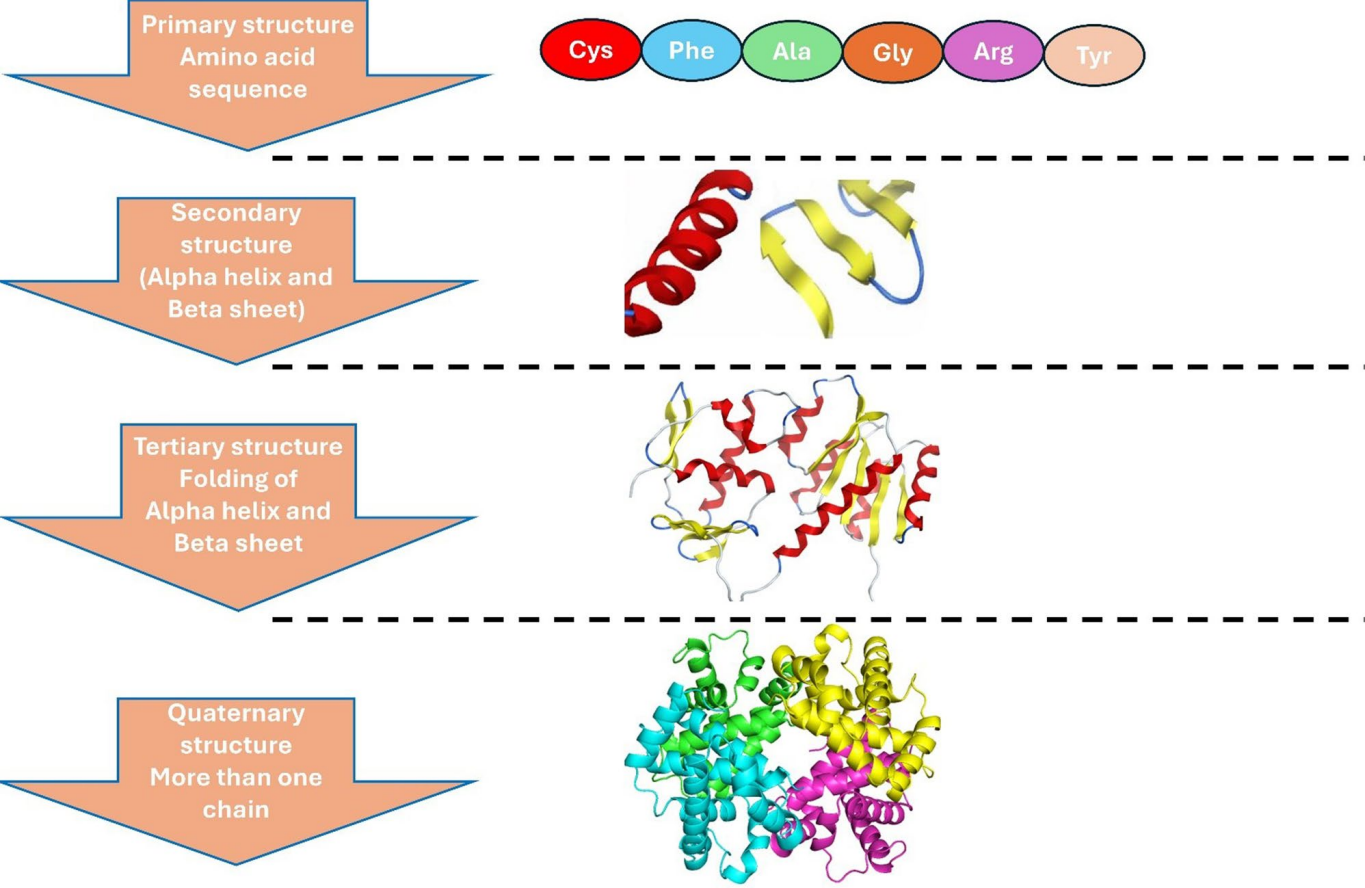
Representation of the primary to quaternary structures of proteins

Protein modeling is a computational prediction of the protein’s three-dimensional structure to understand its function. This can help to reveal the host viral proteins interaction, identifying the active sites that guide in designing inhibitors to block the replication of the virus [85]. Also, it has a significant role in vaccine design by identifying the most potent epitopes that trigger the immune system for viral infectious disease [4]. Proteins are organized into four distinct structural levels, each building on the previous one to create their functional form. The primary structure is the simplest level, consisting of a linear chain of amino acids linked together by peptide bonds. Moving up, the secondary structure introduces local shapes such as alpha-helices and beta-sheets, which are stabilized by hydrogen bonds. The tertiary structure adds complexity by folding the entire polypeptide chain into a unique three-dimensional shape, driven by forces like hydrophobic interactions and disulfide bridges. Finally, the quaternary structure brings everything together, combining multiple protein chains into a larger, functional complex (Fig. 1).

**Fig. 2:**
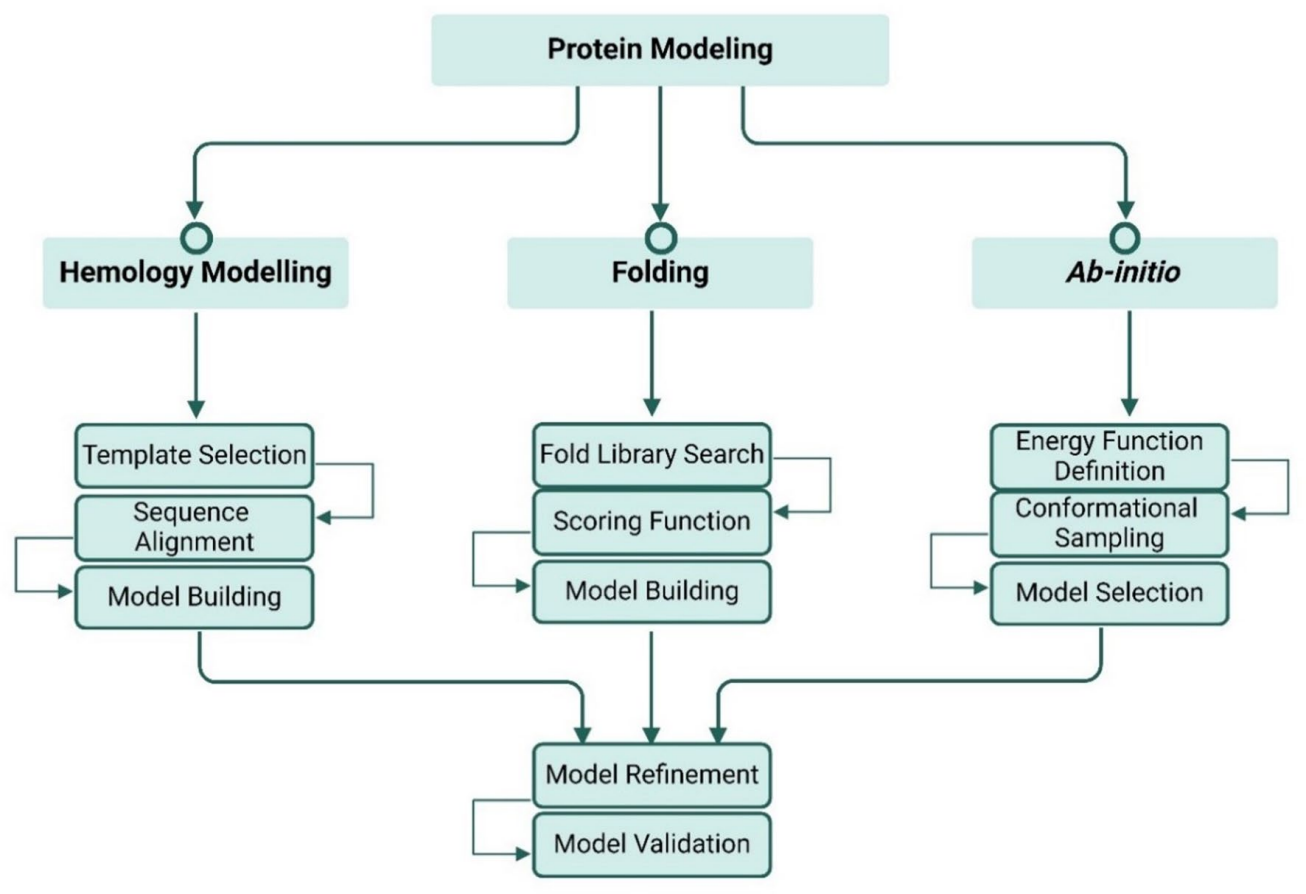
Steps of the three protein modeling methods

There are three primary methods of protein modeling: Homology Modeling, Ab Initio Modeling, and Threading (also known as Fold Recognition). Each method has its unique approach, applications, and restrictions. The homology modeling approach, also known as comparative modeling, depends on the principle that the high similarity of the proteins shares similar structures. This method is based on the availability of templates with 35–50% or higher percent identity or similarity to the target protein sequence [1]. The steps involved in homology modeling are shown in (Fig. 2). Comparative modeling is characterized by high precision when suitable templates are available and quite fast when suitable templates are present. However, this method has challenges once the templates are unavailable or have a low percent identity. Despite these challenges, it plays an important role in drug design and discovery, protein-protein interactions, understanding the functional annotation of proteins [86].

Ab initio modeling, also known as *de novo* modeling, predicts the protein model from scratch when the templates do not exist, as it depends only on the amino acid sequence. This method uses chemical principles and physical fundamentals to identify the conformational space of proteins. The steps involved in ab initio modeling are included in (Fig. 2). Ab initio prediction structure method enables scientists to discover novel and unknown conformational proteins that have not been characterized yet, because of the independence of the template selection. Nevertheless, it needs intensive computational resources, is time-consuming, has less accuracy compared to homology modeling, and is used for small proteins [87].

Threading, also recognized as fold recognition, is used to predict protein when the template similarity is lower than 35%. This method involves the following steps (Fig. 2).

This method performs a comprehensive library of known folded proteins to predict the structure of a candidate protein based on its sequence. However, its accuracy is dependent on the quality and comprehensiveness of this fold library and is generally less precise than homology modeling for targets with a high sequence identity. The folding approach is effective and extensively used because there are few protein folds in native structure because of evolution and also due to the limited imposed with the physics and chemistry of polypeptide chains that verify the resulting protein structure by this technique [88].

Some of the publicly used servers in protein modeling are provided in **(**Table 2**)**. Knowing the principles, advantages, and challenges of the methods helps in choosing the most appropriate approach for target protein modeling requirements, advancing the knowledge of protein structures and their roles in various biological processes.

**Table 2:** List of publicly used servers in protein modeling

Application	Server	Link
3D structure prediction	Robetta	https://robetta.bakerlab.org/submit.php
	Swiss-Model	https://swissmodel.expasy.org/interactive
	I-TASSER	https://zhanggroup.org/I-TASSER/
	AlphaFold	https://colab.research.google.com/github/deepmind/alphafold/blob/main/notebooks/AlphaFold.ipynb
	LOMETS3	https://zhanggroup.org/LOMETS/
	Phyre2	http://www.sbg.bio.ic.ac.uk/~phyre2/html/page.cgi?id=index
	CEthreader	https://zhanggroup.org/CEthreader/
	C-QUARK	https://zhanggroup.org/C-QUARK/
Model Refinement	DeepRefiner	http://watson.cse.eng.auburn.edu/DeepRefiner/index.php
	Galaxy WEB	https://galaxy.seoklab.org/cgi-bin/submit.cgi?type=REFINE
	trRossetta	https://yanglab.nankai.edu.cn/
	Modrefiner	https://zhanggroup.org/ModRefiner/
	PREFMD	http://feig.bch.msu.edu/prefmd
	3Drefine	https://3drefine.mu.hekademeia.org/
	ReFOLD	https://www.reading.ac.uk/bioinf/ReFOLD/
Model Evaluation	SAVES	https://saves.mbi.ucla.edu/
	PROCHECK	https://www.ebi.ac.uk/thornton-srv/software/PROCHECK/
	TM-Score	https://zhanggroup.org/TM-score/
	TM-align	https://zhanggroup.org/TM-align/
	QMEAN	https://swissmodel.expasy.org/qmean/
	Structure Assessment	https://swissmodel.expasy.org/assess

### 3.2. Vaccine Development

Using vaccines is a practical and inexpensive option for preventing or reducing the severity of diseases that impact animals. The traditional vaccination strategy involves culturing the pathogen in a laboratory setting and using it in its weakened or inactivated form to stimulate a protective immune response. On the other hand, purified parts of the pathogen can also be used as antigens in subunit vaccines [89]. Even though not all pathogens can be cultured outside of the body, and the process can be time-consuming, achieving a successful immunization may require an extended period [90].

Remarkably, one of the most important applications of proteomics, especially in veterinary medicine, is vaccine development. Identifying the viral antigens is performed through epitope prediction of the target antigenic protein using different bioinformatics tools **(**Table 3**)** [91]. These epitopes are particularly intriguing in enhancing immunization, as antibodies made from these peptides can enhance or exceed the protective abilities of the original protein [92]. Contrary to live attenuated vaccines, a pathogen’s harmfulness cannot be reversed by an engineered epitope-containing antibody [93]. Moreover, epitope-specific antibodies are highly specific, do not trigger unwanted immune responses, can provide prolonged immunity, and are more cost-effective [94]. The switch in the vaccinology approach involves analyzing the protein groupings of an organism using in silico prediction programs.

**Fig. 3:**
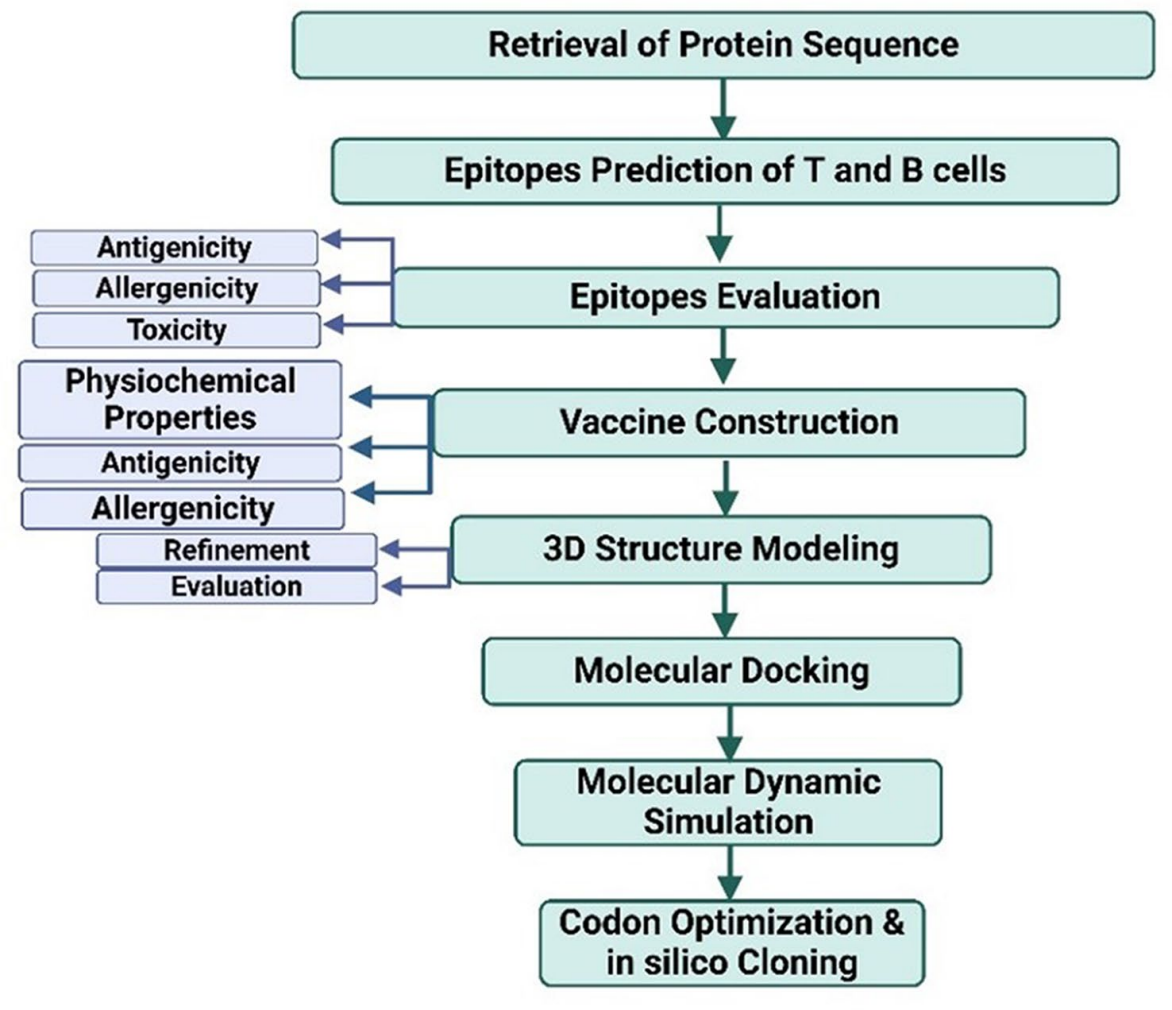
Workflow of vaccine design

After identifying epitopes of the target antigen, the vaccine is constructed using linkers and an adjuvant that connect among the epitopes that prove high antigenicity, non-allergy, and non-toxicity. The steps of the vaccine design are involved in **(**Fig. 3**)**. Additionally, protein modeling aids in designing recombinant proteins derived from different proteins or protein fragments, enabling the usage of these novel proteins to elicit an immune response with broad coverage [95]. Evaluation of the constructed vaccine can also occur firstly in silico through molecular docking and simulation studies of the antibody-antigen interaction, thereby knowing the mechanism of immunogenicity of this constructed modeling vaccine in triggering the immune response [96]. Overall, protein modeling increases the speed of vaccine development by reducing the requirement for extensive experimental testing and enabling a quicker response to emerging viral threats.

Applying the previously mentioned steps for vaccine design was carried out on some viral veterinary infectious diseases such as FMDV serotype O in [9], and for SAT2 in [97], LSDV in [18, 19, 98] and goat pox in [99] that need further experimental studies to validate its efficacy and effectiveness.

**Table 3:** List of publicly used epitopes prediction servers

B cell epitope prediction	SVMTriP	http://sysbio.unl.edu/SVMTriP/prediction.php
	Bepipred Linear Epitope Prediction tool 2.0	https://services.healthtech.dtu.dk/services/BepiPred-2.0/
	Immune Epitope Database and Analysis Resource (IEDB)	http://tools.iedb.org/bcell
	BCEPS	http://imbio.med.ucm.es/bceps/
	ElliPro	http://tools.iedb.org/ellipro/
Prediction of T Cell Epitopes	RANKPEP	http://imed.med.ucm.es/Tools/RANKPEP.html
	SYFPEITHI	http://www.syfpeithi.de/bin/mhcserver.dll/epitopeprediction.htm
	MHCII-NP	http://tools.iedb.org/mhciinp/
	NetMHCpan − 4.1	https://services.healthtech.dtu.dk/services/NetMHCpan-4.1/
	NetMHCIIpan − 4.1	https://services.healthtech.dtu.dk/services/NetMHCIIpan-4.1/

## Transcriptomics application of different veterinary viral infectious diseases

Transcriptomics has a pivotal role in understanding viral infection mechanisms. In gene expression analysis, changes reveal which genes are turned on (upregulated) or turned off (downregulated) during infection and at what time [100]. Building on this knowledge, transcriptomics is used to develop diagnostic biomarkers that can detect specific RNA signatures associated with viral infections. This allows for early diagnosis and detection, which improves control measures. Also, it has a great role in the differential diagnosis of infections caused by similar pathogens such as NDV in poultry [16].

The quick advancement of transcriptome sequencing technology has resulted in the enhancement of single-cell and single-nucleus transcriptome analysis, enhancing sequencing precision and expediting drug discovery. The rise of single-cell transcriptomics has led to the emergence of ST as a related method. Spatial transcriptomics offers data on the levels of expression of genes. This technology has demonstrated great promise in investigating disease mechanisms and finding new drugs [101]. The uses of ST analysis modules continue to expand, with applications solving three fundamental biological problems. First, ST techniques can reveal the cellular composition of tissues, making it possible to observe specific cell types in spatial cellular structures. Second, examining the spatial location and contact between cells facilitates the analysis of cell-cell interactions in tissues. Finally, ST techniques analyze molecular interactions by studying the transcription of ligand-receptor pairs between cells [102].

Transcriptomics helps uncover how viruses establish and maintain PI, shedding light on host immune responses and viral strategies for evasion. A study examines the mechanisms behind persistent FMDV infection in cattle by analyzing gene expression in nasopharyngeal tissues. Researchers compared the transcriptomes of 11 FMDV carriers and 7 non-carriers, finding 648 differentially expressed genes, most upregulated in carriers. Key findings include higher expression of genes related to immune response and cellular proliferation in carriers, while non-carriers showed higher expression of genes promoting virus clearance. The results suggest that FMDV persistence may be due to impaired apoptosis and local suppression of antiviral immunity by regulatory T cells [10]. Another transcriptome analysis of peripheral blood mononuclear cells from PI cattle with BVD revealed 29 differentially expressed genes, including chronic upregulation of interferon-gamma. These findings highlight immune dysregulation in PI cattle, providing insights into how BVD evades host defenses and sustains infection [13].

Additionally, transcriptomics reveals how acute viral infections disrupt host cellular pathways, offering insights for developing targeted therapies. In a study investigating how BVD overcomes host defenses, RNA sequencing identified thousands of differentially expressed genes at different infection stages (2, 6, 12, and 24 h). Key findings included the upregulation of genes involved in lipid metabolism and the downregulation of antiviral response genes like ISG15 and Mx1. These changes suggest that BVD suppresses the host’s immune system while exploiting metabolic pathways to support viral replication [103]. Research on BoHV-1 infection in dairy calves compared transcriptomes of infected and control groups, identifying 488 differentially expressed genes. Key pathways, such as cytokine-receptor interactions and NOD-like receptor signaling, were significantly enriched. These findings highlighted genes involved in viral defense and inflammation, offering potential therapeutic targets for managing BoHV-1-induced respiratory disease [104].

What’s more, transcriptomics supports vaccine development by identifying key antigens and evaluating immune responses post-vaccination. In vaccine studies for CPV, transcriptomics revealed distinct gene expression profiles in vaccinated versus unvaccinated animals after viral challenge. The findings helped identify viral antigens triggering strong immune responses, leading to improved vaccine formulations [105].

Advanced transcriptomics techniques, such as single-cell and spatial transcriptomics, offer insights into cellular interactions and disease mechanisms. Transcriptomic analysis of RABV-infected salivary glands identified key genes and pathways, such as PI3K-Akt signaling, involved in viral pathogenesis. A separate study on RABV-infected mouse brains revealed 22,079 differentially expressed genes associated with immune responses and oxidative phosphorylation. These findings provided new targets for rabies control and treatment strategies [14].

In general, transcriptomics provides comprehensive insights into host-pathogen interactions, diagnostic biomarkers, vaccine development, and therapeutic strategies that help to control and prevent viral diseases in animals, improving animal health and welfare.

## Metabolomics application of different veterinary viral infectious diseases

Metabolomics is the comprehensive study of metabolites, the small molecules produced during metabolism, within a biological system [106]. It is a powerful tool for understanding the biochemical changes that occur during viral infections such as shifts in amino acid utilization, increased energy demands, and alterations in lipid metabolism. It provides insights into disease mechanisms, identifies biomarkers for diagnosis, and informs the development of new treatments and preventive measures. In terms of importance, metabolomics is highly significant for identifying potentially druggable metabolic proteins and/or metabolic regulators through advanced flux analysis, such as through the interpretation of metabolic pathways [107].

However, metabolomics faces challenges, including the complexity of multivariate data analysis [108]. Techniques like PCA and PLS-DA are frequently employed for data visualization and pattern grouping, but careful validation methods are necessary to avoid data overfitting [109]. Despite these challenges, metabolomics remains instrumental in advancing veterinary research across diagnostics, therapies, and vaccine development.

Viruses manipulate host metabolism to create a favorable environment for replication and survival. By uncovering these virus-induced changes, metabolomics identifies potential therapeutic targets [110]. Integration study of metabolomic and transcriptomic of swine testicular cells infected with TGEV revealed significant metabolic disruptions. Pathways such as nucleotide metabolism and bile secretion were altered, with downregulated metabolites linked to bile secretion. The addition of DCA, a key metabolite, enhanced TGEV replication via NF-κB and STAT3 signaling pathways. These findings highlight DCA’s role in facilitating viral replication, offering new avenues for therapeutic interventions [111]. Also, in chicken embryo fibroblasts infected with MDV, metabolomic analysis identified changes in 261 metabolites, particularly in amino acid and energy metabolism. The early stages of infection showed an upregulation of amino acids, which the virus likely exploited to fuel its replication. These results provide a deeper understanding of MDV-host interactions and suggest potential targets for disease control [20].

Furthermore, metabolomics excels at identifying specific metabolites as biomarkers, enabling early and accurate diagnosis of infections [112]. For instance, chickens infected with IBDV, metabolomic profiling identified 368 altered metabolites, including amino acids and glycerophospholipids. These changes, enriched in pathways like tryptophan and lipid metabolism, revealed distinct metabolic disruptions caused by IBDV. Such biomarkers not only enhance understanding of the host response but also support the development of diagnostic tools and therapeutic targets [113]. Another case study on Johne’s Disease, a 17-month longitudinal study monitored calves infected with MAP. Metabolomic analysis revealed key metabolites, such as isobutyrate and branched-chain amino acids, which differentiated infected from non-infected animals. These findings demonstrated metabolomics’ sensitivity in detecting early MAP infections, surpassing conventional diagnostic tools [114].

Moreover, metabolomics helps evaluate how vaccines and drugs influence host metabolism, enabling researchers to improve formulations and ensure safety [115]. Metabolomic studies on COVID-19 have shown how the virus disrupts lipid, amino acid, and carbohydrate metabolism to facilitate replication. These findings provided critical insights for developing treatment strategies and designing more effective therapeutic approaches [116]. In beef steers with BRD, plasma metabolomic analysis revealed distinct differences in metabolites like sarcosine and methionine, which were higher in healthy steers. These metabolites emerged as biomarkers for BRD, aiding in disease diagnosis and management strategies to improve cattle health [117].

In brief, Metabolomics is a key tool in veterinary viral disease research, aiding in the development of diagnostics, vaccines, and treatments to improve livestock health and control viral outbreaks.

## Metagenomics application of different veterinary viral infectious diseases

Metagenomics studies genetic material recovered directly from environmental samples, providing a comprehensive view of the microbial communities present, including viruses, bacteria, fungi, and other microorganisms [118]. It plays a transformative role in identifying pathogens. Sequencing all the genetic material in a sample enables the discovery of novel viruses that might otherwise go undetected [119]. Unlike traditional diagnostic methods that often focus on a single pathogen, metagenomics provides a comprehensive profile of all viruses present in a sample, facilitating the understanding of mixed infection (co-infection) and the complex interactions among multiple pathogens [17]. Also, it offers valuable epidemiological insights by tracking viral diversity and abundance across populations and environments, helping to identify reservoirs, transmission routes, and factors driving viral evolution [119].

Bioinformatics is integral to advancing molecular diagnostics by identifying key issues and developing innovative solutions [120]. For example, bioinformatics has been used to discover antigenic epitopes in the rabies virus glycoprotein G for subunit vaccine development [121] and to identify potential inhibitors targeting the LSDV-encoded DNA polymerase protein, such as taxifolin, which shows promise for LSDV therapeutics [122].

In one study, whole genome metagenomic profiling explored the microbiome community of LSDV pox lesions of infected animals. The findings showed considerable differences in bacterial communities across skin samples, suggesting that specific microorganisms play unique roles in each host. It also highlights the challenges of choosing the right bioinformatics tools for analyzing metagenomic data in clinical and veterinary settings, which makes it an important foundation for future research into how these viruses cause disease [17]. Another study from South Korea examined neurological symptoms in 32-day-old broiler chickens. Conventional diagnostic methods identified *Staphylococcus* spp. in the liver and heart but failed to detect the underlying cause. Metagenomic analysis of brain samples revealed Pseudomonas spp. and MDV both linked to chicken meningoencephalitis. This finding underscores the limitations of traditional diagnostic methods and demonstrates the power of metagenomics in identifying unexpected or unknown pathogens [123].

Metagenomics also plays a crucial role in studying emerging threats. In Italy, canine fecal samples were analyzed using metagenomics, leading to the discovery of the first reported canine kobuvirus and sapovirus. These viruses were associated with gastroenteric diseases in dogs, showing the role of metagenomics in identifying pathogens that might go undetected with conventional methods [124]. Similarly, metagenomic sequencing of brain tissues from sheep with encephalitis revealed a novel astrovirus, linking the virus to the neurological symptoms and highlighting the value of metagenomics in uncovering the causes of unexplained clinical conditions [125].

Additionally, viral metagenomics is invaluable for studying tick-borne viruses. It provides insights into viral diversity, transmission dynamics, and evolution, enabling researchers to predict and prepare for future outbreaks [126]. In conclusion, metagenomics is a powerful tool for uncovering novel pathogens, understanding complex infections, and advancing diagnostic and therapeutic strategies.

## Challenges in multi-omics integration

Integrating multi-omics data is a powerful approach that enables a deeper understanding of viral diseases, but its broader application faces significant challenges. Among these, the disparity between human and animal data in public databases stands out. Public repositories such as NCBI, ENSEMBL, and UniProt have prepared vast amounts of data available for human pathogens and their interactions with hosts, driving innovation in diagnostics, therapeutics, and vaccine development. However, similar databases for veterinary species are sparse or incomplete, creating a gap that limits the translation of cutting-edge discoveries from human to animal health.

This discrepancy underscores the critical role of public databases in advancing our understanding of infectious diseases [21]. For humans, comprehensive datasets enable large-scale comparative studies, detailed evolutionary analyses, and the identification of biomarkers for disease detection and treatment. In contrast, for animals, the lack of robust datasets often restricts researchers to narrow scopes, reducing their ability to perform cross-species analyses or understand zoonotic transmission risks.

High expenses also pose a challenge, especially in veterinary science, where research funding often lags behind that for human medicine. Sequencing technologies, computational infrastructure, and the manpower required for multi-omics studies are costly, deterring efforts to create and maintain databases specifically for veterinary pathogens. This lack of resources perpetuates the reliance on human-centric databases, even though zoonotic diseases frequently emerge from animal populations [127].

Species-specific variations further complicate multi-omics integration. While human databases provide standardized annotations and pathways, the same cannot be said for many animal species. Variations in drug metabolism, immune responses, and host-pathogen interactions across species demand more tailored approaches, making it difficult to directly apply human-derived insights to veterinary contexts [128].

To bridge these gaps, it is vital to prioritize the development of veterinary-specific public databases. Collaborative initiatives between academia, industry, and government agencies can pool resources and expertise to address this need. Furthermore, integrating human and animal datasets into unified platforms can provide a more comprehensive view of zoonotic diseases, facilitating earlier detection and intervention. Ultimately, building robust public databases for veterinary multi-omics will not only benefit animal health but also enhance our preparedness for emerging zoonotic threats, safeguarding both human and animal populations. By recognizing the interdependence of human and veterinary health, we can foster a more integrated approach to combating infectious diseases.

## Ethical considerations and data privacy

The integration and sharing of multi-omics data also raise significant ethical concerns, particularly in international collaborations. The issue of data protection is paramount, requiring secure storage and transfer methods to safeguard sensitive genetic and animal health information. Additionally, ensuring privacy is crucial, especially in cases involving proprietary research or international partnerships, where breaches could lead to misuse of data [129]. There is also the need for equitable use of shared datasets, necessitating clear guidelines to prevent exploitation or unethical applications of the data. Moreover, promoting transparency in data sharing while respecting intellectual property rights and ethical standards is essential for fostering trust among collaborators [127]. Developing comprehensive ethical frameworks and promoting global cooperation will be key to addressing these concerns effectively and ensuring the responsible use of multi-omics data in research and practice.

## Conclusion

This review highlights the transformative potential of omics technologies in advancing veterinary viral research. By exploring distinct omics methodologies and their integration, researchers can gain a comprehensive understanding of viral diseases, which paves the way for innovative diagnostics, vaccines, and therapeutic strategies. Looking ahead, future research should focus on several key areas. First, addressing integration challenges by developing robust tools and workflows that manage data compatibility, standardization, and computational complexity. Second, expanding ethical frameworks to ensure data privacy, equitable use, and ethical collaboration in global research. Third, enhancing real-world applications by translating multi-omics findings into actionable strategies for diagnostics, vaccine development, and disease control in veterinary medicine. Finally, exploring emerging technologies, such as single-cell and spatial omics, to uncover novel insights into host-pathogen interactions. By addressing these challenges and research gaps, the field can continue to make significant progress in veterinary medicine, ultimately improving animal health and welfare globally.

## Data Availability

No datasets were generated or analysed during the current study.

## Abbreviations

NGS: Next-generation sequencing
WGS: Whole genome sequencing
HTS: High-throughput sequencing
SMRT: Single Molecule Real-Time
cDNA: Complementary DNA
RNA-Seq: RNA Sequencing
BAM: Binary Alignment/Map
SAM: Sequence Alignment/Map
VCF: Variant call file
VEP: Variant Effect Predictor
IGV: Integrative Genomics Viewer
MSA: Multiple sequence alignment
FastQC: Fast Quality Control
MultiQC: Multi-quality Control
Trimmomatic: Trimming Tool for Illumina Sequence Data
Cutadapt: Adapter Trimming Tool for NGS Data
BWA: Burrows-Wheeler Aligner
Bowtie2: Fast and Memory-efficient Short-read Aligner
STAR: Spliced Transcripts Alignment to a Reference
Minimap2: Fast Genome Mapping and Alignment Tool
GATK: Genome Analysis Toolkit
FreeBayes: Bayesian Genetic Variant Detector
Samtools: Sequence Alignment/Map Tools
ANNOVAR: ANnotation of VARiation
SnpEff: SNP (Single Nucleotide Polymorphism) Effect Predictor
UCSC Genome Browser: University of California, Santa Cruz Genome Browser
Robetta: Automated Protein Structure Prediction Server
Swiss-Model: Automated Homology Modeling Tool
I-TASSER: Iterative Threading ASSEmbly Refinement
AlphaFold: Deep Learning-Based Protein Structure Prediction Tool
LOMETS3: Local Meta-threading Server 3
Phyre2: Protein Homology/analogY Recognition Engine Version 2
CEthreader: Contact-threading Protein Prediction Tool
C-QUARK: Contact-guided QUARK Structure Modeling Tool
DeepRefiner: Deep Learning-Based Protein Refinement Tool
Galaxy WEB: Open-source Web-based Workflow Platform
trRosetta: Deep Learning Tool for Protein Structure Prediction Using Rosetta
ModRefiner: High-Resolution Protein Structure Refinement Tool
PREFMD: Protein REFinement and Modeling with Deep Learning
3Drefine: Protein Structure Refinement Tool
ReFOLD: Protein Model Refinement Server
SAVES: Structural Analysis and Verification Server
PROCHECK: Program for Checking Stereochemical Quality of Protein Structures
TM-Score: Template Modeling Score
TM-align: Sequence-independent Protein Structure Alignment Tool
QMEAN: Qualitative Model Energy ANalysis
Structure Assessment: Methods or Tools for Assessing Protein Structure Quality
FMD: Foot and mouth disease
BVD: Bovine viral diarrhea
BTD: Blue tongue disease
ASFV: African Swine Fever Virus
LSDV: Lumpy Skin Disease Virus
MDV: Marek’s Disease Virus
BoHV-1: Bovine Herpesvirus 1
CPV: Canine Parvovirus
TGEV: Transmissible Gastroenteritis Virus
IBDV: Infectious Bursal Disease Virus
MS: Mass spectrometry
GPMDB: Global Proteome Machine Database
NDV: Newcastle Disease Virus
RABV: Rabies Virus
PI: Persistent infections
PCA: Principal Component Analysis
PLS-DA: Partial least squares regression discriminant analysis
DCA: Deoxycholic acid
MAP: Mycobacterium avium subsp. paratuberculosis
BRD: Bovine Respiratory Disease
NCBI: National Center for Biotechnology Information
ENSEMBL: European Bioinformatics Institute’s Genome Browser
UniProt: Universal Protein Resource

## Glossary

Bioinformatics: A discipline that merges biology, computer science, and information technology to analyze biological data, especially genetic sequences.
Multi-Omics: An integrative approach that combines data from various omics fields (such as genomics, proteomics, and metabolomics) to provide a holistic view of biological systems.
Genomics: The study of the complete DNA sequence of an organism, including all its genes and their functions.
Proteomics: The large-scale analysis of proteins, focusing on their functions, structures, and interactions.
Transcriptomics: The examination of all RNA transcripts produced by the genome in specific conditions.
Metabolomics: The comprehensive study of metabolites, which are small molecules involved in metabolism, within a biological system.
Next-Generation Sequencing (NGS): A modern sequencing technology that enables rapid and large-scale DNA sequencing.
Phylogenetic Analysis: The investigation of evolutionary relationships among species based on genetic data.
Homology: The similarity in sequence or structure between different biological entities due to shared ancestry.
Epitopes: Specific regions of an antigen recognized by the immune system, particularly by antibodies.
Variant Calling: The identification of genetic variants (like SNPs) in genomic data obtained from sequenced DNA.
Quality Control (QC): Procedures to ensure that experimental data meets established quality standards.
Read Alignment: The process of matching short DNA sequences produced by sequencing to a reference genome.
Annotation: The identification and labeling of functional elements in a genome, such as genes and regulatory regions.
Molecular Docking: A computational method used to predict how a molecule, such as a drug, interacts with a target protein.
High-Throughput Sequencing (HTS): Techniques that allow for the simultaneous rapid sequencing of large quantities of DNA.
Single-Cell Transcriptomics: A technique that analyzes gene expression at the individual cell level.
Spatial Transcriptomics: A method that provides insights into gene expression within the context of tissue architecture and cellular interactions.
Metagenomics: The study of genetic material obtained directly from environmental samples, allowing for the characterization of microbial communities.
